# Feedrate Fluctuation Minimization for NURBS Tool Path Interpolation Based on Arc Length Compensation and Iteration

**DOI:** 10.3390/mi16040402

**Published:** 2025-03-29

**Authors:** Xing Liu, Pengxin Yu, Haiduo Chen, Bihui Peng, Zhao Wang, Fusheng Liang

**Affiliations:** 1 School of Future Science and Engineering, Soochow University, Suzhou 214400, China; 2262405016@stu.suda.edu.cn; 2 Zhejiang Meishuo Electric Technology Co., Ltd., Yueqing 325600, China; ldsheng1332@163.com (P.Y.); haiduochennb@163.com (H.C.); bihuipengnb@163.com (B.P.); 3 School of Mechanical and Electrical Engineering, Soochow University, Suzhou 215131, China

**Keywords:** CNC machining, feedrate fluctuation, parametric interpolation, NURBS tool path, smooth motion

## Abstract

Real-time parametric interpolation plays a crucial role in achieving high-speed and high-precision multi-axis CNC machining. In the interpolation cycle, the position of the next interpolation point is required to be calculated in real-time to guide the action of the machining process. Due to the existence of the positioning error of the interpolation point, it is extremely difficult to eliminate the feedrate fluctuation, which may lead to dramatic decreases in machining quality and the driving capabilities’ saturation of each axis. A computationally efficient and precise feedrate fluctuation minimization method is proposed for the NURBS tool path interpolation in the CNC milling process. The model for the arc length and curvature, with respect to the parameter of the NURBS tool path, is established to reduce the calculation amount required by interpolation points determination. The deviation between the theoretical and actual interpolation step length is decreased by the proposed arc length compensation method to minimize the feedrate fluctuation. In addition, the interpolation points derived from the arc length compensation process are further corrected by performing the Newton iteration to restrict the feedrate fluctuation within the preset accuracy threshold. The effectiveness and superiorities of the proposed feedrate fluctuation minimization method are verified by simulation and milling experiments.

## 1. Introduction

Precision and ultra-precision multi-axis CNC machining is one of the most effective means to obtain precision parts, such as bearings, molds, and lenses. While extensive research focuses on enhancing machining precision through equipment upgrades or novel machining methods [1,2], such approaches often escalate the processing complexity and costs substantially. It is an economical and reliable solution to enhance the manufacturing capacity of the existing machining equipment by improving the processing techniques [3,4] or optimizing processing parameters [5,6]. Specifically, the machining accuracy and quality can be significantly enhanced through systematic optimization of critical factors such as tool path geometry smoothing [7,8], feedrate planning [9], and advanced interpolation algorithms. Among these optimization strategies, tool path geometry smoothing primarily focuses on enhancing the continuity and smoothness of the machine tool movements, whereas feedrate optimization is specifically designed to maximize the machining efficiency while maintaining precision. Despite ensuring optimal smoothness in both the tool path geometry and feedrate profiles, persistent feedrate fluctuations inevitably occur due to discrepancies between the actual and theoretical interpolation step lengths, ultimately leading to the decreased machining stability. Such a feedrate fluctuation not only has a profound impact on machining accuracy and quality, but also affects the service life of the machining equipment. Therefore, investigating the mechanisms and mitigation strategies for the interpolation-induced feedrate fluctuations is of great significance in enhancing the overall stability and reliability of CNC machining processes.

In the interpolation process, the interpolation accuracy and real-time performance are the main indicators used to measure the interpolation performance of a CNC system, which is closely related to the high-speed and high-precision machining process. The feedrate-guided parametric interpolation enables rapid computation of both interpolation step lengths and subsequent tool positions, thereby significantly enhancing the real-time performance of CNC interpolation processes. For most parametric tool paths, especially the currently widely employed non-uniform rational B-Splines (NURBS) tool path, the precise analytical relationship between the arc length and parameters cannot be obtained. Thus, the approximation methods, such as the first-order and second-order Taylor expansion methods, are adopted to achieve the parametric tool position corresponding to the interpolation step length. This approximation introduces systematic errors that predominantly account for feedrate fluctuation in the interpolation process of CNC systems. The feedrate fluctuation, manifested as the deviation between the actual processing speed and the theoretical feedrate, may cause the drastic changes in cutting force and the saturation of the driving capabilities of each axis, which will reduce the machining precision and surface quality [10]. Therefore, it is necessary to develop an accurate and efficient calculation method to reduce the feedrate fluctuation while ensuring the real-time interpolation of the CNC system.

The acquisition of parameter increments corresponding to the interpolation step size through approximation methods constitutes an effective approach for obtaining interpolation positions. The Taylor expansion method represents a well-established and efficient technique that utilizes higher-order derivatives to approximate the increment of the dependent variable. This method demonstrates particularly high approximation accuracy within the domain of small increments of the independent variable. Consequently, Taylor expansion-based interpolation has been widely used as a general and effective parametric interpolation method. Since the interpolation accuracy of the Taylor expansion method is closely related to the interpolation period and the Taylor expansion order, it is necessary to reasonably set the two parameters to limit the interpolation error within an allowable range. Although the Taylor expansion method is not advantageous in terms of interpolation accuracy and real-time performance, it is still widely used in motion control systems, such as CNC machine tools and robots, due to its excellent versatility [11]. Considering the impact of excessive computational complexity of NURBS tool path evaluation and derivation on the real-time performance of interpolation, the Taylor expansion method is usually combined with other methods to achieve simultaneous improvement in interpolation accuracy and computational efficiency. Liu et al. [12] proposed a polynomial equation-based interpolation method to determine the parameter increment in the interpolation process of the NURBS tool path. The Taylor expansion method is used to accelerate the evaluation and derivation of NURBS. However, the employment of Newton’s method greatly affects the real-time performance of the interpolation. A cosine theorem-based double interpolation algorithm was proposed by Wang et al. [13] to obtain an accurate parameter increment in a real-time interpolation module. In one interpolation period, the second-order Taylor’s expansion is performed two times to improve the interpolation accuracy. The application of the composite method is indeed beneficial as a means to improve the interpolation accuracy, but the inherent truncation error of the Taylor expansion method is unavoidable. Thus, it is necessary to fundamentally innovate the parametric interpolation method.

The above approximation-based approach employed for interpolation point position calculation has proven to be insufficient in satisfying the demanding accuracy requirements and real-time performance of modern high-precision CNC machining systems. In order to reduce the computational complexity in real-time interpolation, the predictor–corrector interpolation for the parametric tool path is developed. Furthermore, Baek et al. [14] proposed a NURBS interpolator in which a proportional difference equation was adopted to simply calculate the parameter increment by making full use of the recursive characteristics of NURBS. Without any derivation on NURBS, this method can achieve high interpolation accuracy and reduce the feedrate fluctuation accordingly. Based on the chord length–parameter ratio, Hu et al. [15] adopted the Newton’s divided difference interpolation polynomial to estimate the temporary next parameter value; then, the initial parameter was corrected to reduce the interpolation error. By employing a second-order Runge–Kutta method and a parameter compensation process, the next interpolation parameter can be approximatively achieved with high computational efficiency [16,17,18]. In addition, by combining the co-lateral triangle deviation (CTD) algorithm and Taylor expansion method, the interpolation error can be accurately compensated to achieve minimal feedrate fluctuation [19]. Similarly, a quintic polynomial prediction algorithm is proposed to estimate the target length in one interpolation period based on the historical interpolation knowledge [20]. To improve the convergence rate in the interpolation parameter correction process, a Steffensen iterative accelerator method is developed by Jiang et al. to update the parameters’ value repeatedly [21,22]. Nie et al. [23] developed a two-level parameter compensation method to address the issue of feedrate fluctuation. Although some progress has been made in mitigating feedrate fluctuations, these methods still rely on Taylor series expansions for parameter correction.

The construction of an accurate model for the arc length parameterization of NURBS toolpaths holds significant importance for enhancing interpolation precision. Moreover, determining interpolation points based on the established functional relationship between the parameter and arc length can effectively improve the computational efficiency of real-time interpolation. Among the commonly employed approaches, arc length parameterization methods and arc length parameter fitting techniques are widely utilized. However, precise arc length parameterization methods are not universally feasible for all tool paths [24]. In contrast, the parameter–arc length fitting is a more reliable and versatile method for parametric interpolation [25]. Lei et al. [26] calculated the arc length of the NURBS tool path using an adaptive numerical quadrature method based on the Simpson criterion, and constructed the inverse arc length function (ILF) of the NURBS tool path and the parameter through cubic Hermite spline fitting. In real-time interpolation, the parameter increment corresponding to the interpolation step length can be directly calculated by ILF without any complex derivation and iterative calculation of NURBS. To further reduce the feedrate fluctuation, the calculation of the parameter increment is converted into solving a quartic equation by performing Taylor series expansion on the numerator and denominator of the NURBS tool path, respectively [27]. Moreover, polynomial expression of the parameter–arc length mapping is conducive to quickly and accurately deriving the parameter value corresponding to the interpolation step to reduce the feedrate fluctuation [28], such as the sampled data fitting with the cubic [29] and quintic [30] spline. It has been revealed that the feedrate fluctuation in the parametric interpolation of this parameter–arc length fitting method is smaller than that of the second-order Taylor expansion method, and has stronger robustness [31].

Even though the Taylor expansion interpolation method can enhance interpolation accuracy by increasing the expansion order, it is inevitably constrained by truncation errors and the deterioration of computational efficiency associated with higher expansion orders, which limit its application in high-precision real-time interpolation. Furthermore, predictor–corrector methods and arc length parameterization approaches struggle to simultaneously achieve both interpolation accuracy and computational efficiency. It is thus necessary to emphasize that even if an exact correspondence between the arc length of the NURBS toolpath and the parameter can be established, fluctuations in feedrate remain unavoidable. To address the aforementioned issues, an arc length compensation and Newton iteration-based interpolation method is proposed. Initially, we conducted a rigorous theoretical derivation to analyze the causes of feedrate fluctuations and pre-established piecewise B-spline fitting models based on the arc length–parameter and curvature–parameter relationships of the NURBS toolpath, thereby laying a solid foundation for both interpolation accuracy and real-time performance. Subsequently, to address the issue of feedrate fluctuations during parametric interpolation, an arc length compensation method was proposed to mitigate the deviation between the actual interpolation chord length and the toolpath arc length. Furthermore, considering the high-precision requirements for interpolation parameter positions, the Newton iteration method was employed to refine the interpolation step length. Finally, a series of simulation cases and experiments were conducted to validate the effectiveness of the proposed feedrate fluctuation constraint method.

## 2. Causes of Feedrate Fluctuation in the Parametric Interpolation Process

In order to reduce the computational burden in the real-time parametric interpolation process, the feedrate is usually pre-calculated offline. In each interpolation cycle, the position of the next interpolation point is calculated based on the parametrized feedrate curve *v*(*u*) and the NURBS tool path ***C***(*u*) with the consistently synchronized parameter *u*. Assuming that the parameter of the current interpolation point is *u_i_,* the step length ∆$s_{i}^{*}$ within the interpolation period *T_s_* is as follows:

(1) $$\Delta s_{i}^{*}=v_{i}^{*}T_{s}$$

where $v_{i}^{*}$ is the theoretical feedrate at the interpolation point *u_i_*, which can be calculated directly from the feedrate curve *v*(*u*). In the interpolation process, the actual feedrate ${\hat{v}}_{i}$ can be derived by

(2) $${\hat{v}}_{i}=\frac{\Delta s_{i}}{T_{s}}=\frac{\Delta s_{i}}{\Delta u_{i}}\frac{\Delta u_{i}}{\Delta s_{i}^{*}}\frac{\Delta s_{i}^{*}}{T_{s}}=\underset{\alpha }{\underset{︸}{\left\|C^{\prime }(u_{i})\right\|\frac{\Delta u_{i}}{\Delta s_{i}^{*}}}}\;\;\cdot v_{i}^{*}$$

where ∆*u_i_* is the actual interpolation parameter increment; ∆*s_i_* is the corresponding actual interpolation step length; *α* is the proportional coefficient between ${\hat{v}}_{i}$ and $v_{i}^{*}$; and $\left\|C^{\prime }(u_{i})\right\|$ is the arc differential of the NURBS tool path at the interpolation point *u_i_*. If the actual interpolation parameter increment ∆*u_i_* is inconsistent with the parameter increment corresponding to the theoretical interpolation step length ∆*s_i_*, the coefficient *α* in Equation (2) is not equal to 1. Under these circumstances, there is a deviation between the theoretical feedrate $v_{i}^{*}$ and the actual feedrate ${\hat{v}}_{i}$, and the feedrate fluctuation occurs. Since it is difficult to determine the analytical relationship between the arc length *s* of the NURBS tool path and the parameter *u*, the parameter increment ∆*u_i_* corresponding to the interpolation step length ∆$s_{i}^{*}$ can only be derived by approximation methods, such as the Taylor series and the Runge–Kutta method. Due to the existence of the truncation error in the approximate method, it is bound to cause estimation errors in parameter increments, which inevitably causes feedrate fluctuation. The feedrate fluctuation can be defined by

(3) $$\varepsilon =\left|\frac{v_{i}^{*}-{\hat{v}}_{i}}{v_{i}^{*}}\right|\times 100\%,\;\;\;\;\;\;\;\;\;\;\;{\hat{v}}_{i}=\frac{\overset{⏜}{C_{i}C_{i+1}}}{T_{s}}$$

where $\overset{⏜}{C_{i}C_{i+1}}$ is the actual interpolation step length (arc length increment from point ***C***(*u_i_*) to point ***C***(*u_i_*_+1_) along the tool path); *u_i_*_+1_ is the next interpolation parameter point in actual processing; and *u_i_*_+1_ = *u_i_* + ∆*u_i_*.

**Figure 1:**
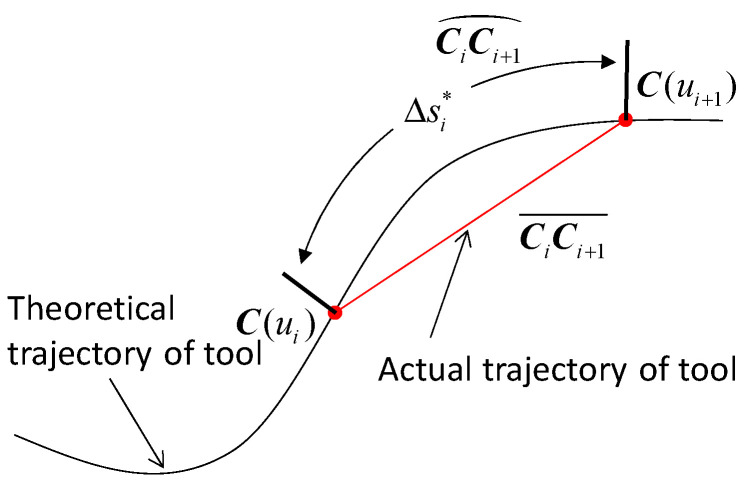
The motion trajectory of tool in CNC milling process.

In the CNC milling process, the motion trajectory of the tool moves in a straight line from the current interpolation point ***C***(*u_i_*) to the next point ***C***(*u_i_*_+1_) instead of moving along the arc length direction of the tool path, as shown in Figure 1. Assuming there is no deviation in the estimation of the parameter increment ∆*u_i_*, the arc length $\overset{⏜}{C_{i}C_{i+1}}$ is definitely the theoretical interpolation step length ∆$s_{i}^{*}$. When the machining trajectory is the chord line $\bar{C_{i}C_{i+1}}$, the feedrate can be derived by

(4) $${\hat{v}}_{i}=\frac{\left\|C(u_{i+1})-C(u_{i})\right\|}{T_{s}}$$

This means that even if there is no deviation in the estimation of ∆*u_i_*, the feedrate fluctuation still exists. Such a feedrate fluctuation can be rewritten as follows:

(5) $$\varepsilon =\left|\frac{v_{i}^{*}-{\hat{v}}_{i}}{v_{i}^{*}}\right|\times 100\%=\left|\frac{\Delta s_{i}^{*}-\left\|C(u_{i+1})-C(u_{i})\right\|}{\Delta s_{i}^{*}}\right|\times 100\%$$

Without considering the estimation error of the parameter increment ∆*u_i_*, the feedrate fluctuation mainly comes from the deviation between the theoretical interpolation step length ∆$s_{i}^{*}$ ($\overset{⏜}{C_{i}C_{i+1}}$) and the chord length $\bar{C_{i}C_{i+1}}$. This deviation can only be completely eliminated when the machining tool path is a straight line. On the contrary, in the high curvature area of the NURBS tool path, this deviation and the resulting feedrate fluctuation will become particularly obvious. As shown in Equation (5), if the parameter deviation of ∆*u_i_* is not taken into consideration, the actual feedrate ${\hat{v}}_{i}$ will not be greater than the theoretical feedrate $v_{i}^{*}$, since $\bar{C_{i}C_{i+1}}$ ≤ ∆$s_{i}^{*}$.

When the scheduled feedrate cannot ensure that the machining process along the segmented tool path is completed within an integer that is multiple of that of the interpolation period, the displacement discarded in the rounding process needs to travel in an additional interpolation period [32]. This unmatched running displacement in the last interpolation period of each segmented tool path unit inevitably causes serious feedrate fluctuation. In contrast to the two aforementioned causes of feedrate fluctuation, this issue can be completely eliminated during the feedrate planning process [33,34]. Therefore, in the real-time interpolation process, the restriction of such feedrate fluctuation is not considered in this study.

## 3. Feedrate Fluctuation Restriction Method

### 3.1. Compensation of Arc Length in the NURBS Tool Path Interpolation

**Figure 2:**
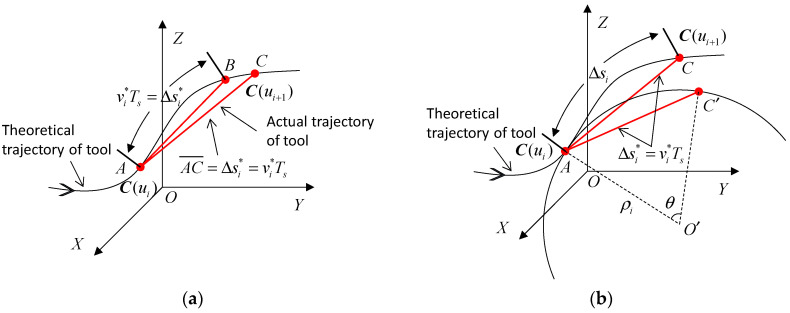
Parametric interpolation with the compensation of arc length. (**a**) Adjustment of chord length; (**b**) adjustment of arc length.

The parametric interpolation process is presented in Figure 2a. To reduce the feedrate fluctuation caused by the deviation between the actual interpolation chord length and the arc length of tool path, the position of the next interpolation point is supposed to fall at point C instead of point B, and the chord length is $\bar{AC}$= $v_{i}^{*}$T_s_. On this occasion, the actual feedrate ${\hat{v}}_{i}$ is the same as the theoretical feedrate $v_{i}^{*}$, and the deviation between the actual interpolation chord length and the arc length of tool path is eliminated.

It is a complicated process to directly determine a point *C* on the tool path ***C***(*u*) to make the actual interpolation chord length $\bar{AC}$ equal to the theoretical interpolation step length ∆$s_{i}^{*}$($v_{i}^{*}$T_s_). As shown in Figure 2b, assuming that the curvature radius at the current interpolation point ***C***(*u_i_*), i.e., the point *A*, is *ρ_i_*, the approximate arc length $\overset{⏜}{AC}$ corresponding to the chord length $\bar{AC}$ can be calculated by the circular arc approximation method. An arc *O*′ with a radius of *ρ_i_* at the current interpolation point *A* is created, and the chord length $\bar{AC^{\prime }}$ on the arc *O*′ is equal to the chord length $\bar{AC}$ on the tool path. *θ* is the central angle corresponding to the chord length $\bar{AC^{\prime }}$. The arc length $\overset{⏜}{AC^{\prime }}$ on the arc *O*′ corresponding to the chord length $\bar{AC^{\prime }}$ can be calculated by

(6) $$\left\{\begin{array}{l} \frac{\left\|\overset{⏜}{AC^{\prime }}\right\|}{\rho_{i}}=\theta \\ \sin(\frac{\theta }{2})=\frac{\left\|\bar{AC^{\prime }}\right\|}{2\rho_{i}}=\frac{v_{i}^{*}T_{s}}{2\rho_{i}} \end{array}\right.$$

Using the above equation, the interpolation step length ∆*s_i_* can be approximately represented by

(7) $$\Delta s_{i}=\left\|\overset{⏜}{AC}\right\|\approx \left\|\overset{⏜}{AC^{\prime }}\right\|=\rho_{i}\theta =2\rho_{i}\arcsin(\frac{v_{i}^{*}T_{s}}{2\rho_{i}})$$

Taking the first two terms of the Maclaurin expansion of the inverse sine function to approximately replace the inverse sine term in Equation (7), the following equation can be derived:

(8) $$\Delta s_{i}\approx 2\rho_{i}\left[\frac{v_{i}^{*}T_{s}}{2\rho_{i}}+{\left(\frac{v_{i}^{*}T_{s}}{2\rho_{i}}\right)}^{3}\right]=v_{i}^{*}T_{s}+\frac{{\left(v_{i}^{*}T_{s}\right)}^{3}}{24{\rho_{i}}^{2}}$$

In parametric interpolation, the interpolation step length along the tool path in the current interpolation cycle is taken as ∆*s_i_* instead of ∆ $s_{i}^{*}$. In this manner, the actual interpolation chord length $\bar{AC}$ will be close to the theoretical interpolation step length. Since the compensation within a single interpolation period is minimal (typically less than 1 mm), employing the arc approximation method to compensate for the interpolation step length can still achieve high interpolation accuracy, even when applied to three-dimensional spatial toolpaths. Thus, the feedrate fluctuation caused by the deviation between the interpolation chord length and interpolation arc length can be effectively limited.

However, the aforementioned arc length compensation method requires further discussion for trajectories with an excessively large or small curvature radius. When the curvature radius approaches positive infinity, Equation (7) contains $\arcsin(\frac{v_{i}^{*}T_{s}}{2\rho_{i}})\approx \frac{v_{i}^{*}T_{s}}{2\rho_{i}}$, and, thus, it can be corrected as

(9) $$\Delta s_{i}\approx 2\rho_{i}\frac{v_{i}^{*}T_{s}}{2\rho_{i}}=v_{i}^{*}T_{s}$$

This indicates that when the curvature radius is too large, the arc length compensation is ineffective. On the other hand, when the curvature radius approaches zero, it is evident that the above equation no longer holds, meaning that this arc length compensation method is not applicable for cases with a very small curvature radius. Therefore, it is necessary to impose the following restrictions on the use of this method:

(10) $$\rho_{i}\geq v_{i}^{*}T_{s}$$

After obtaining the new interpolation step length ∆*s_i_* with the arc length compensation method, it is necessary to introduce approximation methods, such as Taylor expansion or *s*-*u* fitting, to calculate the parameter increment corresponding to the arc length increment ∆*s_i_* of the NURBS tool path, and to obtain the next interpolation parameter point *u_i_*_+1_. In order to reduce the calculation amount in the interpolation process, the *s*-*u* fitting method is adopted to procure the functional relationship between the arc length and parameter of the NURBS tool path to realize real-time interpolation. The function of the arc length with respect to the parameter is written as $\hat{s}=g(u)$, where $\hat{s}$ is the normalized arc length. Additionally, the function of the parameter with respect to the arc length is written as $u=f(\bar{s})$, where $\bar{s}$ is the normalized arc length parameter. In the arc length compensation process, the curvature of the interpolation points on the NURBS tool path needs to be determined. The curvature calculation in real-time interpolation will be simplified by constructing a functional relationship between the curvature and parameter to avoid the second-order derivative calculation of the NURBS tool path. The function of curvature with respect to the parameter is written as *k* = *φ*(*u*), where *k* is the curvature of NURBS tool path.

### 3.2. Feedrate Fluctuation Restriction Based on the Newton Iteration Method

The parametric interpolation method with arc length compensation ensures high interpolation accuracy. However, if further reduction in feedrate fluctuation is required, additional correction must be applied to the interpolated results. Consequently, the Newton iteration method is adopted to perform interpolation point correction, thereby ensuring strict control over feedrate fluctuations. The parameter of the interpolation point that is derived directly from the arc length compensation process is used as the initial value of the iteration. The deviation between the actual feedrate and the theoretical feedrate is gradually reduced by iteratively adjusting the interpolation point, so that the feedrate fluctuation is less than the given precision threshold *ε*_max_. The NURBS tool path interpolation process based on arc length compensation and the Newton iteration method is as follows:

Step 1: The arc length of the NURBS tool path at the current interpolation point is represented by *s_i_* = *s_N_* × *g*(*u_i_*), where *s_N_* is the total arc length of the NURBS tool path.
Step 2: The theoretical step length of the current interpolation cycle is calculated by ∆$s_{i}^{*}$ = $v_{i}^{*}$T_s_.
Step 3: Then, calculate the curvature radius of the NURBS tool path at the current interpolation point *u_i_* using the equation *ρ_i_* = 1/*k_i_ =* 1/*φ*(*u_i_*). The approximate interpolation arc length along the tool path is calculated by ∆*s_i_* ≈ $v_{i}^{*}$T_s_ + ($v_{i}^{*}$T_s_)^3^/24$\rho_{i}^{*}$ using the arc length compensation method.
Step 4: Then, calculate the next interpolation parameter point $u_{i+1}^{0}$ = *f*((*s_i_* + ∆*s_i_*)/*s_N_*) corresponding to the arc length increment ∆*s_i_*.
Step 5: Based on the initial value $u_{i+1}^{0}$, the Newton iteration method is used to calculate the next interpolation point *u_i_*_+1_, at which the feedrate fluctuation is limited to a predetermined threshold *ε*_max_.
Step 6: If *u_i_*_+1_ ≥ 1, the interpolation process terminates; otherwise, *i* = *i*+1, and go to step 1.

The effect of the above iterative parametric interpolation method is closely related to the constructed parameter–arc length and parameter-curvature model of the NURBS tool path, i.e., $\hat{s}=g(u)$, $u=f(\bar{s})$, and *k* = *φ*(*u*). As long as the accuracy of these models is high enough, the precise feedrate fluctuation restriction during the interpolation process can be realized. Therefore, the piecewise B-spline fitting method is employed to acquire high-precision *u*-*s*, *s*-*u,* and *u*-*k* curves, as shown in Appendix A. Even if the Newton iteration method in step 5 is not employed to correct the interpolation parameter, the feedrate fluctuation restriction is still better than that used in the traditional method, such as the second-order Taylor expansion method, by using only the arc length compensation-based interpolation method. In addition, the evaluation and derivation calculation of the NURBS tool path is not required in this method; therefore, the real-time performance is very high.

## 4. Implementations

**Figure 3:**
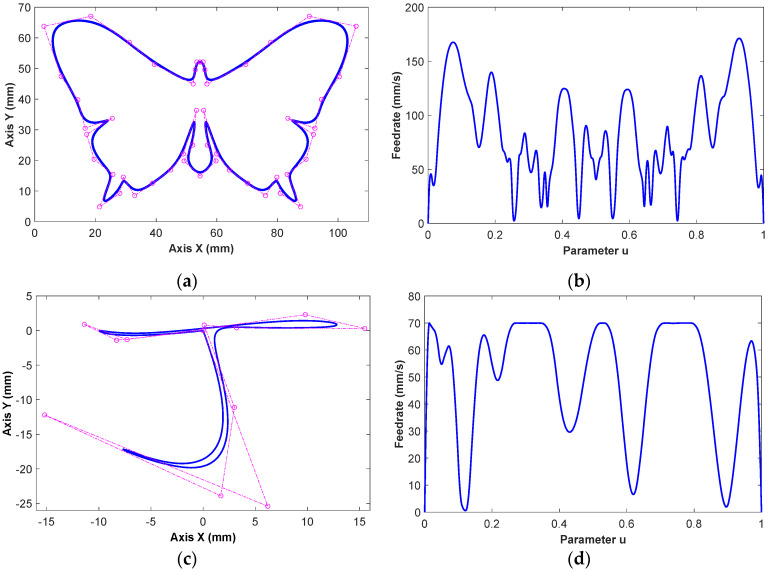
NURBS tool paths and B-spline ffeedrate. (**a**) NURBS tool path and (**b**) feedrate guide curve for Case I, and (**c**) NURBS tool path and (**d**) feedrate guide curves for Case II.

Two typical NURBS tool paths are employed to verify the effectiveness of the proposed feedrate fluctuation restriction method. As shown in Figure 3a,c, each NURBS tool path has multiple curvature features, which seriously affects the feedrate fluctuation in the interpolation process. The time-optimal feedrate guide curves are derived by using a genetic optimization algorithm-based method [10]. As shown in Figure 3b,d, the feedrate is represented by the smooth three-degree B-spline curves. The feedrate constraints are the same as those shown in the previous research [35]. A comparative analysis with Taylor expansion and *s*-*u* fitting methods is conducted to exhibit the superiority of the proposed method in feedrate fluctuation restriction.

### 4.1. Case I

**Figure 4:**
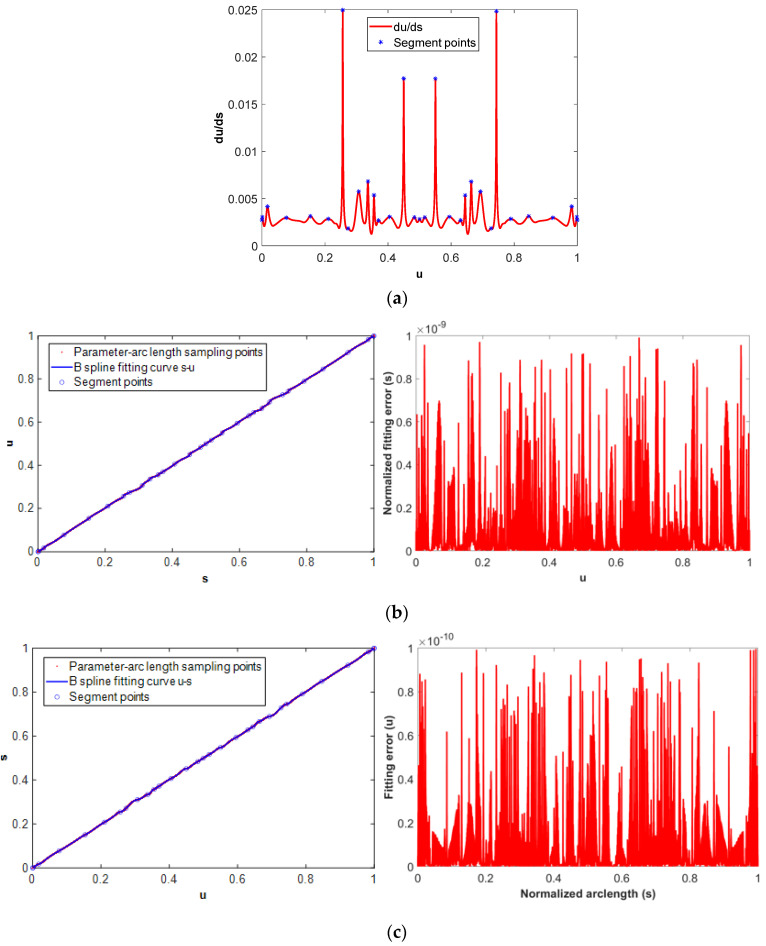
B-spline fitting of parameter–arc length sampling points. (**a**) Parameter–arc length segment points, (**b**) B-spline fitting of *s*-*u* curve and its associated fitting error, (**c**) B-spline fitting of the *u*-*s* curve and its associated fitting error.

The arc length of the butterfly-shaped NURBS tool path as shown in Figure 4a is calculated by numerical integration method, and the arc length–parameter discrete points are obtained using sampling method. The initial parameter interval for the arc length numerical integration is set to Δ*u* = 1.0 × 10^−4^ and the arc length integration tolerance is set to *ε_s_*= 1.0 × 10^−16^ mm. The piecewise fitting method is used to fit the discrete points (*u_i_*, *s_i_*) (i = 0~N) into a B-spline curve, where N is the number of sampling points. The derivative of parameter with respect to the arc length of the NURBS tool path, i.e., *du*/*ds*, is shown in Figure 4a. The fitting of the parameter–arc length discrete sampling point is segmented at the maximum point of *du*/*ds*. As shown in the blue point of Figure 4a, 31 maximum points divide the sampling points into 30 segments. Figure 4b,c are the B-spline fitting results of the *s*-*u* and *u*-*s* sampling points. The fitting accuracy of the *s*-*u* curve is 1.0 × 10^−9^, and the fitting accuracy of the *u*-*s* curve is 1.0 × 10^−10^.

**Figure 5:**
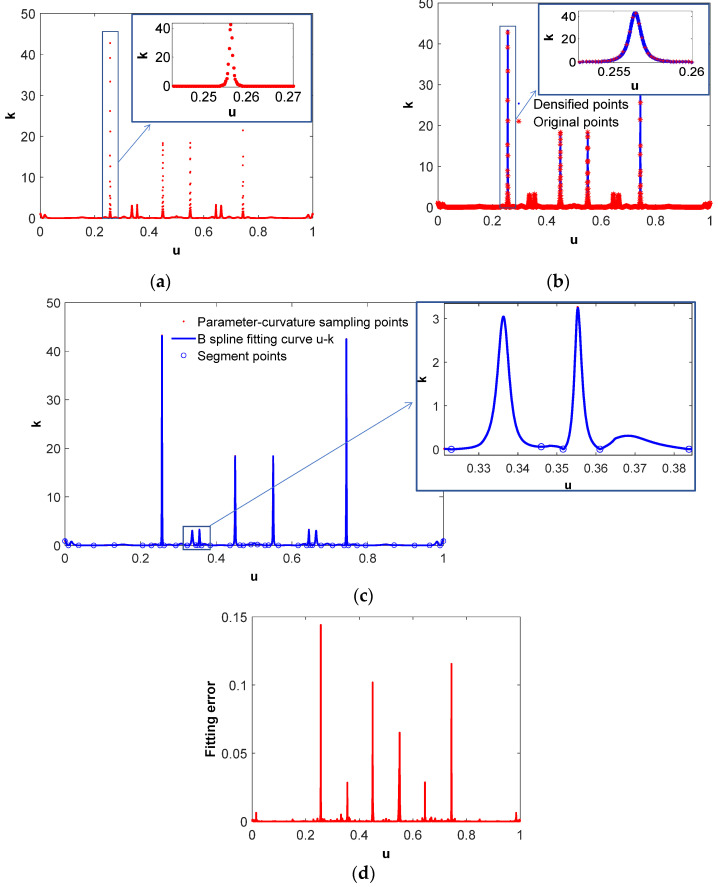
B-spline fitting of parameter-curvature sampling points. (**a**) Original parameter-curvature sampling points, (**b**) densified parameter-curvature sampling points, (**c**) B-spline fitting of the *u*-*k* curve, (**d**) fitting error of the *u*-*k* curve.

When sampling the parameter-curvature (*u*-*k*) of the NURBS tool path, the initial sampling interval is set to Δ*u* = 2.0 × 10^−4^. The discrete sampling points obtained according to the initial sampling interval is shown in Figure 5a. In the area where the curvature changes significantly, the sampling points are too sparse to cover all curvature information. Figure 5b shows the densified *u*-*k* sampling points that are evenly distributed on the entire parameter range and can contain almost all the curvature information. The discrete *u*-*k* sampling points are reconstructed into a continuous curve using a piecewise B-spline fitting method, in which the fitting error at each point is limited individually to 0.01 times (one percent) the curvature value of that point instead of setting a uniform accuracy threshold. Figure 5c shows the B-spline fitting results of the sampling points (*u_i_*, *k_i_*) (*i* = 0~*N*), and the fitting error distribution is shown in Figure 5d.

**Figure 6:**
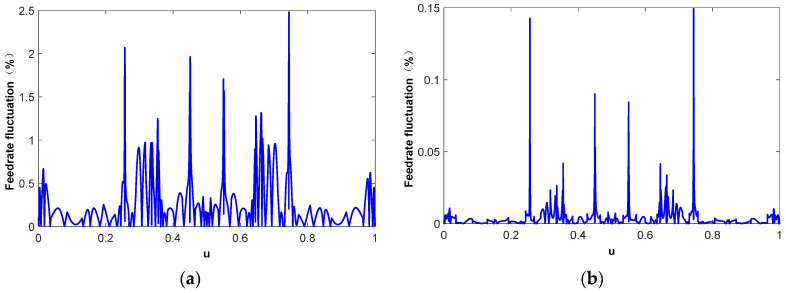

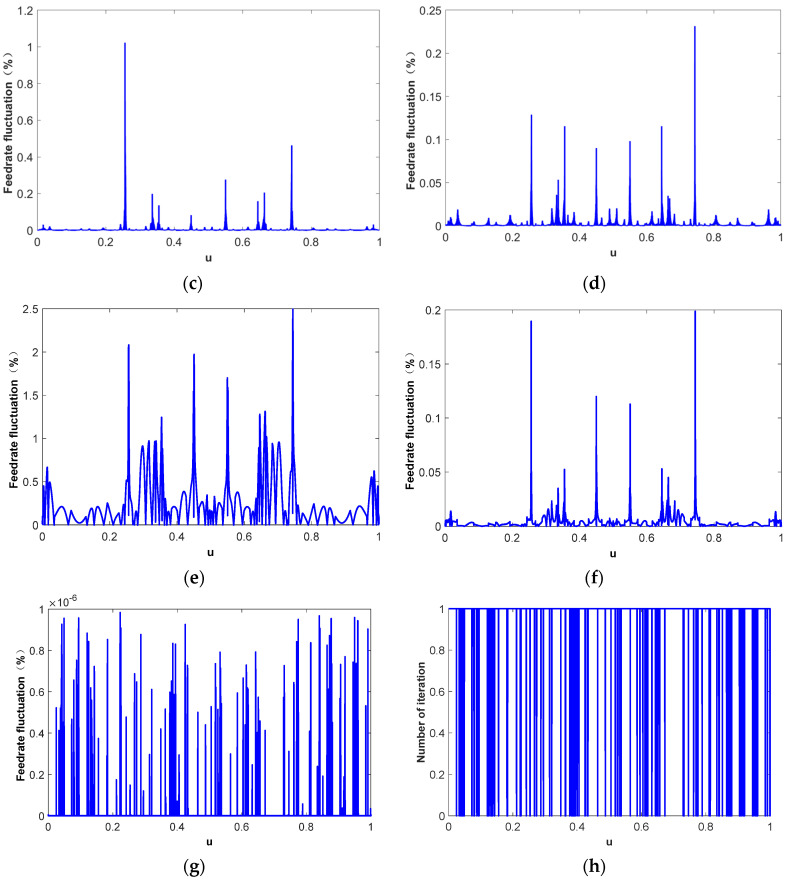
Parametric interpolation results. (**a**) First-order Taylor expansion method, (**b**) second-order Taylor expansion method, (**c**) interpolation based on quintic B-spline fitting curve [36,37], (**d**) interpolation based on 7th-order polynomial fitting curve [7,29], (**e**) arc length compensation-based first-order Taylor expansion method, (**f**) arc length compensation-based second-order Taylor expansion method, (**g**) Newton iteration method based on the initial value derived from *s*-*u* fitting curve and arc length compensation (threshold 10^−6^%), (**h**) number of Newton iteration.

Figure 6 shows the feedrate fluctuation in different parametric interpolation methods. The maximum, mean, and mean square error of the feedrate fluctuation are listed in Table 1. Although the first-order Taylor expansion method has high computational efficiency and excellent real-time performance, it will cause a large feedrate fluctuation. As shown in Figure 6a, the maximum feedrate fluctuation is 2.47%. Figure 6b shows the parametric interpolation results using the second-order Taylor expansion method. Compared to that in the first-order Taylor expansion method, the interpolation accuracy is greatly improved. The maximum feedrate fluctuation is reduced to 0.15% and the reductions in the mean value and mean square error (MSE) are even more significant. However, the second-order Taylor method needs to calculate the second-order derivative of the NURBS tool path, which leads to a poor real-time interpolation performance. Figure 6c shows the interpolation results based on the quintic B-spline fitting curve. The interpolation accuracy and computational accuracy are greatly improved compared to those in the Taylor expansion method, and the interpolation real-time performance is even better than that in the first-order Taylor expansion method. The distribution of feedrate fluctuation is consistent with the curvature distribution of the NURBS tool path, as shown in Figure 5a. This is mainly because the motion trajectory deviation between the theoretical interpolation step length and the actual chord length is positively correlated with the curvature of the NURBS tool path. By employing higher-order polynomial representations, the expressive capability of the curve is enhanced, and the fitting performance is significantly improved. In the case of the 7th-order polynomial fitting method, the overall feedrate fluctuation is effectively constrained, with the maximum feedrate fluctuation limited to within 0.25%. Furthermore, the distribution of feedrate fluctuations across the entire parameter domain becomes more uniform, as illustrated in Figure 6d. In order to explore the effect of arc length compensation on the Taylor expansion interpolation process, the feedrate fluctuation in the first-order and second-order Taylor expansion interpolation based on arc length compensation is drawn in Figure 6e,f. The results indicate that arc length compensation exhibits a negligible impact on the first-order Taylor expansion interpolation method. From a holistic perspective, the overall trend of feedrate fluctuations across the entire parameter space remains largely unchanged, primarily because the correction introduced by arc length compensation on the interpolation chord length is significantly smaller than the truncation error inherent in the first-order Taylor expansion method. However, when the order of the Taylor expansion is increased to the second-order, the effect of arc length compensation gradually becomes apparent, albeit with a certain degree of randomness. This is attributed to the fact that the truncation error of the Taylor expansion can be either positive or negative. Consequently, arc length compensation is not a well-suited complement to the Taylor expansion, and may even lead to inferior results. Generally, the feedrate fluctuation in the CNC milling process needs to be restricted within the range of 0.001–0.1%; however, as can be observed from Figure 6a–f, neither the Taylor expansion-based method nor the fitting-based method alone can reliably restrict the fluctuation within the required limits, as certain portions exceed the threshold. In our proposed approach, we introduce the arc length compensation method, in addition to the s-u fitting method, and utilize it as the initial value for the Newton iteration method to achieve further optimization. The results demonstrate that the feedrate fluctuation is strictly limited to be within 1.0 × 10^−6^%, as illustrated in Figure 6g. Figure 6h shows the number of iterations of the Newton iteration method. The results demonstrate that, at most, one iteration is needed to make the feedrate fluctuation meet the accuracy requirements.

### 4.2. Case II

**Figure 7:**
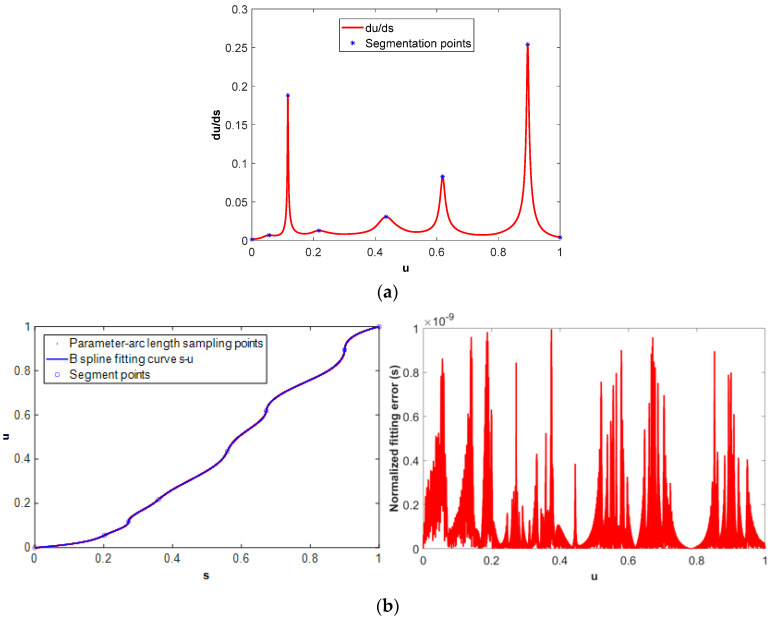

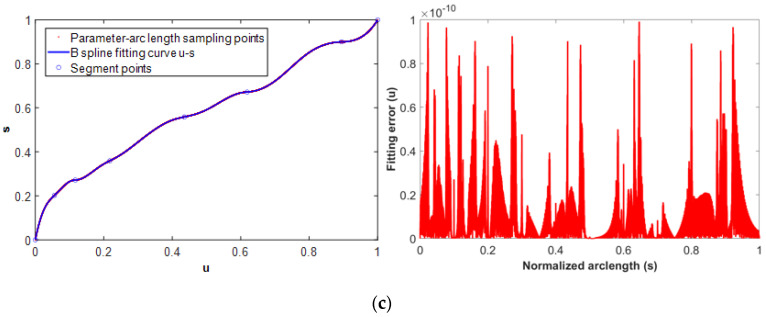
B-spline fitting of parameter–arc length sampling points. (**a**) Parameter–arc length segment points, (**b**) B-spline fitting of *s*-*u* curve and its associated fitting error, (**c**) B-spline fitting of the *u*-*s* curve and its associated fitting error.

**Figure 8:**
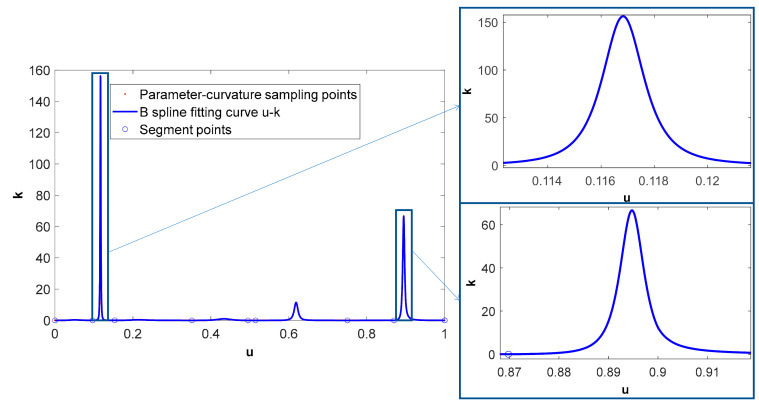
B-spline fitting of parameter-curvature sampling points.

The NURBS tool path, as shown in Figure 3c, has obvious characteristics of drastic changes in curvature and corresponding changes in the B-spline feedrate curve, as shown in Figure 3d. The same initial parameter sampling interval and chord length integration tolerance as those in Case I are used to obtain the parameter–arc length discrete points. The B-spline segmented fitting accuracy for the *s*-*u* curve is 1.0 × 10^−9^, and the fitting accuracy of the *u*-*s* curve is 1.0 × 10^−10^. Figure 7 shows the segmentation points of the tool path in the parameter–arc length fitting process, and the B-spline fitting results of the *s*-*u* curve and the *u*-*s* curve. A total of eight segmentation points divide the tool path into seven segments for fitting. Figure 8 shows the B-spline fitting results of the parameter-curvature sampling points of the tool path. The sampling and fitting accuracy of the parameter-curvature (*u*-*k*) are the same as those in Case I.

**Figure 9:**
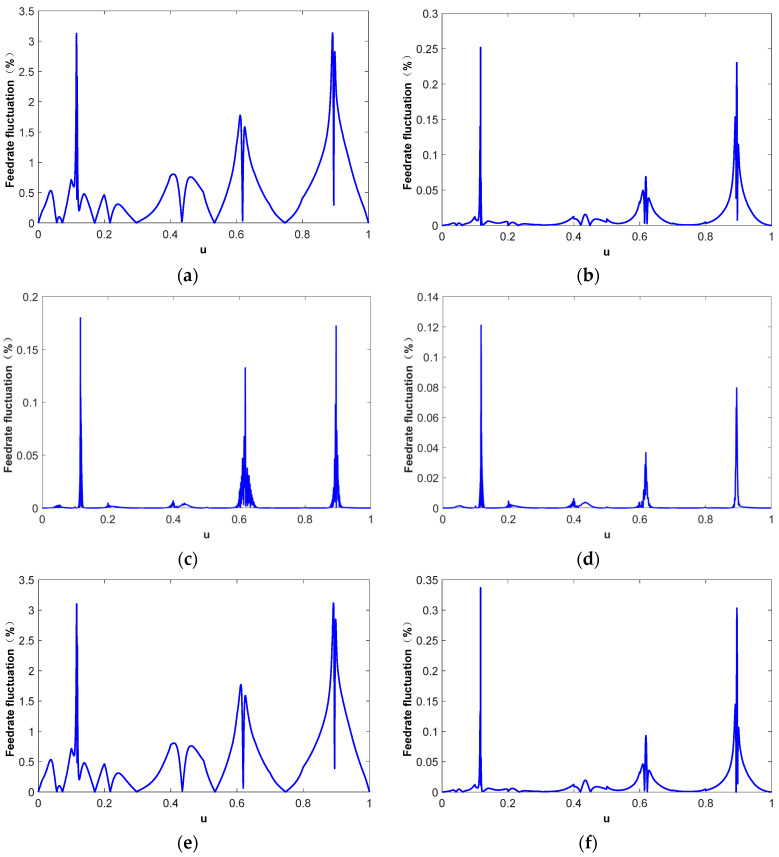

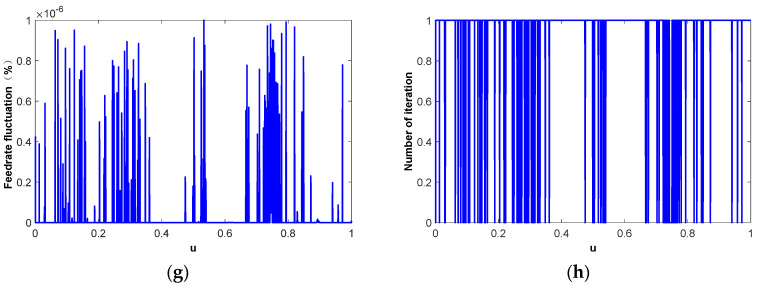
Parametric interpolation results. (**a**) First-order Taylor expansion method, (**b**) second-order Taylor expansion method, (**c**) interpolation based on quintic B-spline fitting curve [36,37], (**d**) interpolation based on 7th-order polynomial fitting curve [7,29], (**e**) arc length compensation-based first-order Taylor expansion method, (**f**) arc length compensation-based second-order Taylor expansion method, (**g**) Newton iteration method based on the initial value derived from *s*-*u* fitting curve and arc length compensation (threshold 10^−6^%), (**h**) number of Newton iteration.

Figure 9 shows the interpolation results using different parametric interpolation methods. The corresponding feedrate fluctuations are shown in Table 2. Since the curvature of the tool path is unevenly distributed on the entire parameter range, the feedrate fluctuation is more obvious in the area near the high curvature. Even though the feedrate limitation in this case is smaller than that in Case I, the maximum feedrate fluctuation is much higher due to the larger maximum curvature of this NURBS tool path. At high curvature points, although the interpolation step length is short, the chord length deviation caused by the tool path bending still leads to large feedrate fluctuation. Figure 9a,b show the interpolation results directly derived from the first-order and second-order Taylor expansion method-based interpolation, respectively. The feedrate fluctuation increases rapidly in high curvature areas. Although the second-order Taylor expansion method has improved the interpolation accuracy by an order of magnitude compared to the first-order expansion method, the feedrate fluctuation still exceeds 0.2%. The quintic B-spline fitting method can significantly reduce feedrate fluctuation and limit the maximum value to less than 0.2%, as shown in Figure 9c, while having extremely high interpolation calculation efficiency. As depicted in Figure 9d, the interpolation accuracy can be further improved through 7th-order polynomial fitting. However, this improvement is marginal and comes at the cost of a significantly increased computational load, thereby compromising the real-time performance of the interpolation process. Moreover, similar to Case I, the arc length compensation method exhibits limited effectiveness in constraining feedrate fluctuations within the Taylor expansion method and may even exert a counterproductive influence, as shown in Figure 9e,f. The Newton iteration method is used to correct the interpolation parameter derived from the arc length compensation-based *s*-*u* fitting method under the predefined feedrate fluctuation threshold (1.0 × 10^−6^%). Figure 9g,h show the corresponding interpolation results and the number of iterations. With one iteration at most, the feedrate fluctuation in the parametric interpolation can be restricted within the preset range.

### 4.3. Case III

**Figure 10:**
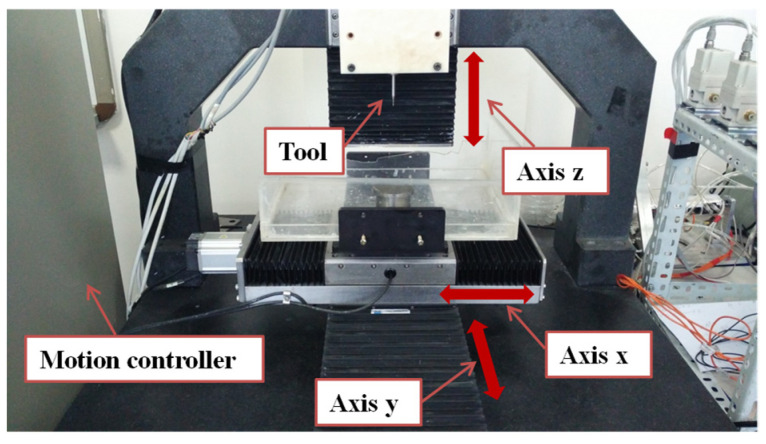
CNC machine tool with open motion controller.

In order to further verify the influence of the proposed method on the interpolation real-time performance and machining accuracy, a milling experiment was carried out on a CNC machine tool, as shown in Figure 10. The experimental platform adopts an open motion controller based on a personal computer, and a programmable multi-axes controller (PMAC) from Delta Tau, which has a high-performance digital signal processor core that can realize the real-time calculation of the NURBS tool path interpolation. The custom interpolation programs can be imported into the PMAC to perform real-time motion control on the machine tool, with the interpolation period configured as 8 ms. The *x*-axis, *y*-axis, and *z*-axis of the experimental platform are all driven by Panasonic’s servo drives and motors. The linear motion is achieved by a screw–nut pair with a pitch of 1 mm. The actual position of the three axes is fed back through the grating ruler, and the maximum resolution of the position feedback is 0.1 μm. The measured positioning precision of each drive axis of the experimental platform is 2 μm. The dynamic performance has been optimized by adjusting the PID parameters.

**Figure 11:**
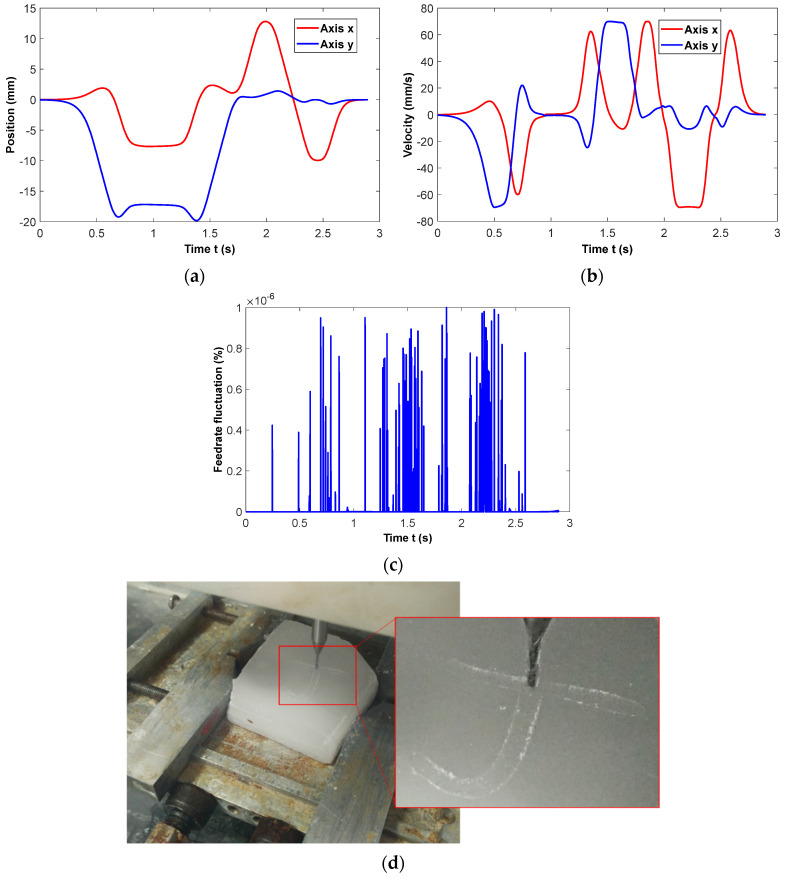
Milling experiment results. (**a**) Motion of *x*-axis and *y*-axis, (**b**) velocity of *x*-axis and *y*-axis, (**c**) feedrate fluctuation, (**d**) milling trajectory.

The milling experiments were conducted following the NURBS tool path and the corresponding B-spline feedrate in Case III, as shown in Figure 3c,d. In order to ensure the real-time performance of interpolation, the maximum number of iterations of the Newton iteration method is limited to no more than one under the condition of the preset feedrate fluctuation threshold *ε*_max_ =1.0 × 10^−6^%. The initial value in the Newton iteration is determined based on the combination of *s*-*u* fitting and the arc length compensation method. The interpolation program is compiled and imported into PMAC for real-time interpolation processing in the milling experiments. The position and velocity of each drive axis are recorded and preserved, as shown in Figure 11a,b; it indicates that the velocity of all drive axes is smooth, without any sudden changes. The actual feedrate fluctuation at the milling cutter tip is calculated and shown in Figure 11c. Under the premise of real-time interpolation, the feedrate fluctuation can be restricted within the preset threshold. Considering the low rigidity of the machine tool spindle, paraffin wax is selected for milling to avoid possible damage to the machine tool structure caused by the high-speed feed motion in the cutting process. A double-edged milling cutter with a diameter of *D* = 2 mm is selected, and the spindle speed is set to 3000 r/min. The total machining time is less than 3 s, and the machining results are shown in Figure 11d. The figure demonstrates that the milling path remains smooth throughout the whole parameter range, even in the transition area with high curvature. The simultaneous improvement of the machining efficiency and machining quality not only benefits from the optimization of the feedrate under high-order constraints, but also lies in the effective restriction of feedrate fluctuation.

**Figure 12:**
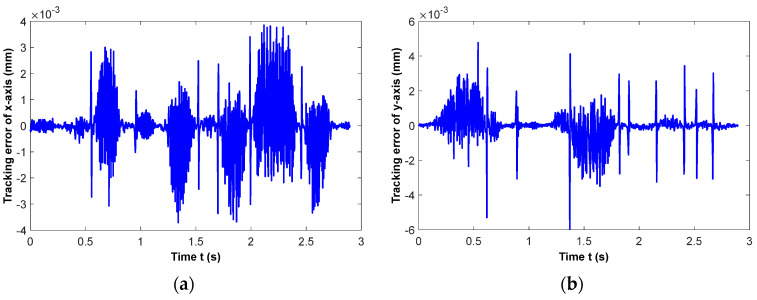
Tracking error of drive axes, (**a**) tracking error of *x*-axis, (**b**) tracking error of *y*-axis.

The tracking errors of the *x*-axis and *y*-axis are recorded and saved, respectively, as shown in Figure 12a,b. Due to the smooth machining process and precise feedrate correction during interpolation, the tracking error of the drive axes can be limited within ±10 μm, which demonstrates that the movement of each axis is limited within the driving capacity. However, in some areas along the machining tool path, the tracking error is still large. The distribution of tracking error fluctuation is basically consistent with that of feedrate fluctuation, as shown in Figure 11c. It indicates that the feedrate stability in the interpolation process is one of the decisive factors affecting multi-axis machining precision.

## 5. Conclusions

Feedrate fluctuation in the real-time parametric interpolation process is the main factor that leads to the precision decrease in multi-axis CNC machining. An arc length compensation combined with the Newton iteration method is proposed to restrict the feedrate fluctuation and to obtain a smooth machining process. This method is capable of effectively restricting the feedrate fluctuation while reducing the calculation amount of real-time NURBS tool path interpolation. The main contributions of this article are drawn as follows.

(1) An effective and computationally efficient arc length compensation method combined with Newton iteration is proposed to reduce the feedrate fluctuation.
(2) The high-precision *s-u-*, *u-s-,* and *u*-*k*-segmented B-spline fitting method is employed to obtain the arc length increment corresponding to the parameter increment and the curvature of tool path at the interpolation points with a small calculation amount.
(3) A comparison with different parametric interpolation methods verifies the superiority of the proposed feedrate fluctuation restriction method.
(4) The milling experiment results demonstrate that the effective restriction of feedrate fluctuation is conducive to obtain a smooth machining process.

The proposed method exhibits extensive applicability in mitigating feedrate fluctuations during real-time parametric interpolation. Nevertheless, its effectiveness is constrained when processing trajectory segments with curvature radii that are smaller than the interpolation step size, as the arc length compensation technique produced overly large estimation inaccuracies under such conditions. Additionally, it is noteworthy that in order to establish the correspondence between s and u, B-spline curves were employed for fitting, and a more accurate and efficient fitting method is yet to be explored further. Furthermore, to preserve as much curvature information of the NURBS curve as possible, a point densification strategy was adopted. However, densifying the sampling points across the entire parameter space is not an ideal approach. A promising direction for future research lies in developing a targeted densification strategy for regions with rich curvature information.

## Figures and Tables

**Table 1:** Feedrate fluctuation in parametric interpolation.

Parametric Interpolation Methods	Feedrate Fluctuation		
	Maximum	Mean	MSE
First-order Taylor expansion	2.47	0.345	0.348
Second-order Taylor expansion	0.149	5.81 × 10^−3^	9.75 × 10^−3^
*s*-*u* fitting curve	4.83 × 10^−2^	7.74 × 10^−4^	2.95 × 10^−3^
*s*-*u* fitting curve and arc length compensation	5.32 × 10^−3^	1.77 × 10^−4^	3.12 × 10^−4^
Arc length compensation-based first-order Taylor expansion	2.49	0.345	0.348
Arc length compensation-based second-order Taylor expansion	0.199	6.01 × 10^−3^	1.13 × 10^−2^
Newton iteration method based on the initial value derived from *s*-*u* fitting curve and arc length compensation (threshold 10^−6^%)	9.83 × 10^−7^	1.32 × 10^−8^	8.99 × 10^−8^

**Table 2:** Feedrate fluctuation in parametric interpolation.

Parametric Interpolation Methods	Feedrate Fluctuation		
	Maximum	Mean	MSE
First-order Taylor expansion	3.13	0.408	0.507
Second-order Taylor expansion	0.252	8.42 × 10^−3^	2.04 × 10^−2^
*s*-*u* fitting curve	8.59 × 10^−2^	9.07 × 10^−4^	5.36 × 10^−3^
*s*-*u* fitting curve and arc length compensation	9.39 × 10^−3^	1.39 × 10^−4^	5.62 × 10^−4^
Arc length compensation-based first-order Taylor expansion	3.116	0.408	0.507
Arc length compensation-based second-order Taylor expansion	0.337	8.61 × 10^−3^	2.26 × 10^−2^
Newton iteration method based on the initial value derived from *s*-*u* fitting curve and arc length compensation (threshold 10^−6^%)	9.99 × 10^−7^	2.25 × 10^−8^	1.19 × 10^−7^

**Figure A1:**
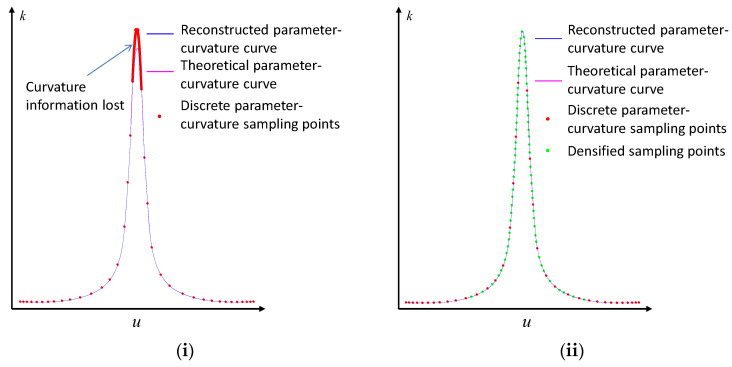
*k_j_* > *k_j_*_−1_; thus, the B-spline *u*-*k* curve with a preset fitting accuracy threshold can be obtained. (**i**) Parameter-curvature sampling with a fixed parameter interval, (**ii**) reconstructed parameter-curvature curve.

## Data Availability

The original contributions presented in the study are included in the article. Further inquiries can be directed to the corresponding author.

## Abbreviations

The following abbreviations are used in this manuscript:

CNC	Computer Numerical Control
NURBS	Non-Uniform Rational B-Splines
CTD	Co-lateral triangle deviation
ILF	Inverse arc length function
PMAC	Programmable multi-axes controller

## Appendix A

### Appendix A.1. B-Spline Fitting for Parameter–Arc Length of NURBS Tool Path

Arc Length Calculation of NURBS Tool Path

For the non-arc length-parameterized NURBS tool path, it is difficult to obtain the exact arc length corresponding to the specified parameter value. The discrete numerical integration method is used to calculate the approximate arc length of the NURBS tool path. The arc length *s_i_* of the NURBS tool path ***C***(*u*) on the parameter range [*u_i,_ u_i_*_+1_] can be derived by

(A1) $$s_{i}=\int_{u_{i}}^{u_{i+1}}\frac{ds}{du}du=\int_{u_{i}}^{u_{i+1}}\left\|C^{\prime }(u)\right\|du=\int_{u_{i}}^{u_{i+1}}f(u)du$$

where

(A2) $$f(u)=\frac{ds}{du}=\left\|C^{\prime }(u)\right\|$$

The Gaussian quadrature rule is used to approximately solve the above equation. The form of the *n*-point Gauss quadrature rule is stated as

(A3) $$\int_{-1}^{1}f(x)dx\approx \sum_{j=0}^{n}A_{j}f(x_{j})$$

where the nodes *x_j_* and weights *A_j_* for *j* = 1, …, *n* can be determined by table lookup. Before applying the Gaussian quadrature rule, the integral over [a, b] must be changed into an integral over [−1, 1] in the following way,

(A4) $$\int_{a}^{b}g(x)dx=\frac{b-a}{2}\int_{-1}^{1}g(\frac{b-a}{2}t+\frac{b+a}{2})dt\approx \frac{b-a}{2}\sum_{j=0}^{n}A_{j}g(\frac{b-a}{2}t_{j}+\frac{b+a}{2})$$

The three-point Gaussian quadrature rule is employed to approximately calculate the arc length of the NURBS tool path. It can be determined that when the following inequality is satisfied,

(A5) $$\frac{1}{10}\left|s(u_{i},\bar{u})+s(\bar{u},u_{i+1})-s(u_{i},u_{i+1})\right|<\varepsilon_{s}$$

where $\bar{u}$ is the midpoint of the integration interval [*u_i_*, *u_i_*_+1_] and *s*(*a*, *b*) is the arc length of the NURBS tool path on the parameter range [*a*, *b*], the following inequality must be true:

(A6) $$\left|\int_{u_{i}}^{u_{i+1}}f(u)du-(s(u_{i},\bar{u})+s(\bar{u},u_{i+1}))\right|<\varepsilon_{s}$$

This means that the Gaussian quadrature error of the arc length over the parameter range [*u_i_*, *u_i_*_+1_] will not exceed the integration tolerance *ε_s_*. Thus,

(A7) $$\int_{u_{i}}^{u_{i+1}}f(u)du\approx s(u_{i},\bar{u})+s(\bar{u},u_{i+1})$$

The integration interval [*u_i_*, *u_i_* + ∆*u*] is specified firstly, where ∆*u* is the initial integration interval. This integration interval is divided into two equal parts, and the arc lengths of each subinterval and the whole interval are obtained by using the Gaussian quadrature rule. If the integration error is limited within the preset integration tolerance *ε_s_*, which can be determined by Equation (e), the integration interval and the corresponding arc length will be marked and kept. Otherwise, each subinterval is re-divided, and the above process is repeated. At last, each subdivided integration interval and the corresponding arc length are reserved, and the discrete parameter–arc length points (*u_i_*, *s_i_*) (*i* = 1~*N*) can be obtained, where *N* is the number of discrete points.

B-spline Fitting on the Discrete Parameter–Arc Length Points for u-s and s-u Curves

In order to fit the discrete parameter–arc length points (*u_i_*, *s_i_*) to the B-spline curve *u*-*s*, the chord length parameterization method is used to parameterize the arc length *s_i_*. Considering the high-precision requirement of the correspondence between arc length and parameters, the number of discrete sampling points (*u_i_*, *s_i_*) is very large. Directly fitting all discrete points with B-spline curves will bring many issues, such as excessive calculation and uncontrollable oscillation during the fitting process. Therefore, it is necessary to divide the original discrete sampling points into small segments, in which the fitting process is performed, respectively. The segmentation principle of discrete points is to minimize the number of oscillations of the first-order derivative of the *u*-*s* curve. Therefore, the maximum value of *du*/*ds* is selected for segmentation, i.e.,

(A8) $$\left\{\begin{array}{l} {\left.du/ds\right|}_{u=u_{j}}>{\left.du/ds\right|}_{u=u_{j-1}}\\ {\left.du/ds\right|}_{u=u_{j}}>{\left.du/ds\right|}_{u=u_{j+1}} \end{array}\right.$$

At this point, *d*^2^*u*/*ds*^2^ is approximately equal to 0. If the above equation is true, (*u_j_*, *s_j_*) will be set as the segment point. In each segmentation interval, the iterative knot insertion (IKI) method [38] is employed to fit the discrete parameter–arc length points (*u_i_*, *s_i_*) to the B-spline curve with a preset fitting accuracy threshold.

By using the piecewise IKI fitting method, not only the *u*-*s* curve $\hat{s}=g(u)$, but also the *s*-*u* curve $u=f(\bar{s})$, can be obtained, where $\bar{s}$ is the parameterized arc length and $\hat{s}$ is the normalized arc length. When using $u=f(\bar{s})$ to calculate the parameter value, the arc length value needs to be divided by the total arc length *s_N_* of the NURBS tool path to convert it into the parameter space. Similarly, when using $\hat{s}=g(u)$ to calculate the arc length value corresponding to the parameter value of the NURBS tool path, the derived normalized arc length needs to be multiplied by *s_N_* to convert it into trajectory space.

### Appendix A.2. B-Spline Fitting for Parameter-Curvature of NURBS Tool Path

Discrete Parameter-Curvature Points Sampling for NURBS Tool Path

When the parameter-curvature (*u*-*k*) points of the NURBS tool path are sampled with a fixed parameter interval Δ*u*, the sampling points will become particularly sparse and cannot contain all curvature information in areas where the curvature changes dramatically. As shown in Figure A1(i), the parameter-curvature sampling with a fixed parameter interval cannot obtain the critical information near the extreme points, which will cause the reconstructed parameter-curvature curve to lose curvature information. Therefore, it is necessary to densify the sampling points near the areas of high curvature. As shown in Figure A1(ii), the sampling points in areas where the curvature is greater than the average value are densified so that the reconstructed parameter-curvature curve can retain all curvature information.
